# Prosaposin down-modulation decreases metastatic prostate cancer cell adhesion, migration, and invasion

**DOI:** 10.1186/1476-4598-9-30

**Published:** 2010-02-04

**Authors:** Siyi Hu, Nathalie Delorme, Zhenzhen Liu, Tao Liu, Cruz Velasco-Gonzalez, Jone Garai, Ashok Pullikuth, Shahriar Koochekpour

**Affiliations:** 1 Stanley S. Scott Cancer Center, School of Medicine, Louisiana State University Health Sciences Center, New Orleans, LA 70112, USA; 2 School of Public Health, School of Medicine, Louisiana State University Health Sciences Center, New Orleans, LA 70112, USA; 3 Departments of Pharmacology, School of Medicine, Louisiana State University Health Sciences Center, New Orleans, LA 70112, USA; 4 Department of Biochemistry and Molecular Biology, School of Medicine, Louisiana State University Health Sciences Center, New Orleans, LA 70112, USA; 5 Department of Microbiology and Immunology, School of Medicine, Louisiana State University Health Sciences Center, New Orleans, LA 70112, USA; 6 Department of Urology, School of Medicine, Louisiana State University Health Sciences Center, New Orleans, LA 70112, USA

## Abstract

**Background:**

Factors responsible for invasive and metastatic progression of prostate cancer (PCa) remain largely unknown. Previously, we reported cloning of prosaposin (PSAP) and its genomic amplification and/or overexpression in several androgen-independent metastatic PCa cell lines and lymph node metastases. PSAP is the lysosomal precursor of saposins, which serve as activators for lysosomal hydrolases involved in the degradation of ceramide (Cer) and other sphingolipids.

**Results:**

Our current data show that, in metastatic PCa cells, stable down-modulation of PSAP by RNA-interference via a lysosomal proteolysis-dependent pathway decreased β_1A_-integrin expression, its cell-surface clustering, and adhesion to basement membrane proteins; led to disassembly of focal adhesion complex; and decreased phosphorylative activity of focal adhesion kinase and its downstream adaptor molecule, paxillin. Cathepsin D (CathD) expression and proteolytic activity, migration, and invasion were also significantly decreased in PSAP knock-down cells. Transient-transfection studies with β_1A _integrin- or CathD-siRNA oligos confirmed the cause and effect relationship between PSAP and CathD or PSAP and Cer-β_1A _integrin, regulating PCa cell migration and invasion.

**Conclusion:**

Our findings suggest that by a coordinated regulation of Cer levels, CathD and β_1A_-integrin expression, and attenuation of "inside-out" integrin-signaling pathway, PSAP is involved in PCa invasion and therefore might be used as a molecular target for PCa therapy.

## Background

Prosaposin (PSAP) is a dual-function highly conserved glycoprotein that exists as the lysosomal precursor of four small sphingolipid activator proteins, known as saposins A, B, C and D [1-3]. Saposins are generated by proteolytic cleavage of another lysosomal protease, cathepsin D (CathD) [4-6]. In lysosomes, mature saposins are intensively involved in metabolism of sphingolipids and ceramide (Cer), functioning either as essential co-factors for sphingolipid hydrolases and/or destabilizing the complex of lipids and membranes [3]. PSAP also exists as a secreted protein, which has been found in various body fluids such as milk, serum, and seminal fluid [2]. Secreted PSAP is a well-known potent neurotrophic factor [7,8]. Total PSAP deficiency is lethal in both man and mice [2]. However, deficiency of individual saposin proteins is responsible for a number of lipid storage diseases [9-11].

Homozygous inactivation of *PSAP *gene in mice led to shrinkage and atrophic changes in the male reproductive organs, with gross pathological features including a reduction in size and weight of the testes, seminal vesicle, and prostate gland [12]. Histological examination of the involuted prostate tissue revealed the presence of undifferentiated epithelial cells. Collectively, these data support a developmental role for PSAP in prostate gland. During our search for a prostate tumor marker, we cloned PSAP as a secreted protein from the highly invasive and metastatic PCa cell line PC-3 [13]. In addition, we discovered its overexpression and/or genomic amplification in several androgen-independent (AI) and/or metastatic PCa cell lines and in punch biopsy samples of LuCaP PCa xenograft and lymph node metastases. Interestingly, PSAP expression in C4-2B, an AI-bone metastatic PCa cell line was significantly higher than in its parental isogenic and marginally tumorigenic cell line, LNCaP [13]. Recently, we demonstrated that saposin C and TX14A-synthetic peptide, two well-known bioactive derivatives of PSAP, act as cell survival and anti-apoptotic factors, stimulate migration and invasion, and activate PI3K/Akt- and MAPK-signaling pathways in PCa cell lines [14-16]. However, the underlying mechanisms of PSAP regulation of PCa cell migration and invasion have not been investigated.

In this study, we evaluated the contribution of PSAP in multistep process of invasion by using an RNA-interference strategy and transient or stable transfectants of metastatic PCa cell lines. Down-modulation of PSAP expression did not alter PCa cell growth. However, by increasing cellular Cer levels and decreasing β_1A_-integrin and CathD expression, PSAP significantly decreased the cell adhesion, migration, and invasion abilities of AI-PCa cells. Taken together, our data support a role for PSAP in invasive and metastatic progression of PCa.

## Results

### PSAP is overexpressed in metastatic PCa cells

**Figure 1:**
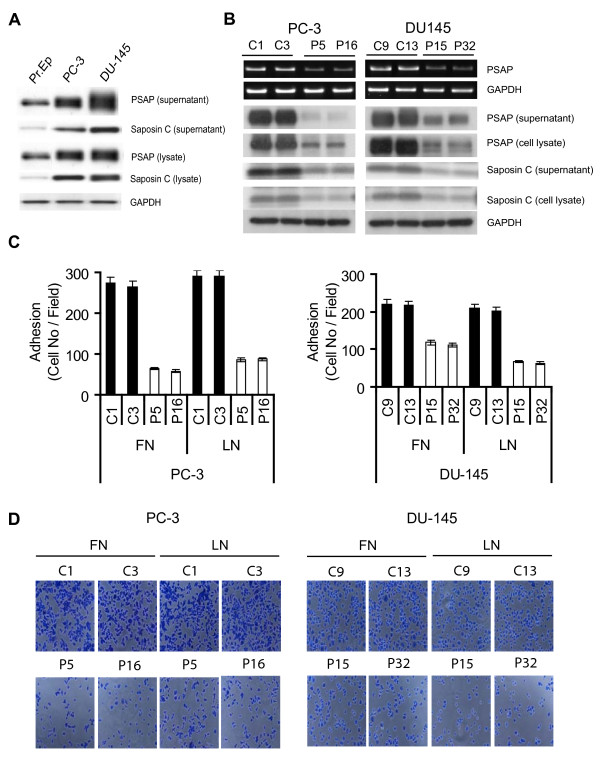
***PSAP *gene silencing decreases metastatic PCa cell adhesion to basement membrane proteins**. (A) PSAP over-expression in metastatic prostate cancer cell lines. Equal amount of cell lysates or supernatants from PC-3, DU-145, and normal prostate epithelial (Pr.Ep) cells were subjected to western blotting with anti-PSAP and saposin C antibodies. (B) Stable PSAP down-modulation was accomplished by short-hairpin RNA targeted at *PSAP *gene. PC-3 and DU-145 metastatic PCa cell lines were stably transfected with a G418-resistant vector containing a shRNA sequence specific for human PSAP or a scrambled control sequence. Total RNA was extracted for RT-PCR *(top)*. GAPDH transcript was used as an internal control for RNA loading. Cell lysates and culture supernatants were subjected to immunoblotting with PSAP antibody. GAPDH antibody was used for protein loading. (C and D) PC-3 and DU-145 cell adhesion to ECM proteins was examined by seeding 1.5 × 10^4 ^cells per well in 96-well plates pre-coated with 10 μg/ml fibronectin (FN) or laminin (LN). After 2 h incubation, adhered cells were fixed and stained with toluidine blue. Cells were photographed and counted from ten random fields at 100 × magnification. *Columns*, mean of three independent samples run together; *bars*, ± SEM, *p *< 0.0001, ANOVA was used to compare PSAP-KD and control clones. Each experiment was repeated three times independently. C1 and C3 in PC-3 and C9 and C13 in DU-145 were control clones (shRNA-scrambled vector) and P5 and P16 in PC-3 and P15 and P32 in DU-145 were PSAP-KD clones (shRNA-PSAP).

As shown in Fig. 1A, PSAP and saposin C are expressed at higher levels in metastatic PCa cell lines than in the normal prostate epithelial cells (Pr.Ep). In addition, using other PCa progression models of isogenic cell lines, we observed consistent data for increased PSAP expression level from normal, poorly tumorigenic, or non-tumorigenic cells to androgen-independent and/or highly invasive and metastatic cell lines such as LNCaP/C4-2B, PC-3/PC-3M, and p69-M12-M2182 (see additional file 1). The biological significances of PSAP as an intracellular or extracellular soluble protein in PCa cells are largely unknown. Our attempts to increase the expression of PSAP in PC-3 and DU-145 cells beyond their endogenous level failed. Therefore, we decided to use RNA interference strategy to specifically down-modulate PSAP expression. After establishing several control or PSAP-KD clones, we randomly selected two clones for each category for further analysis. As shown in Fig. 1B, stable transfection of the two metastatic PCa cell lines with a PSAP-shRNA vector decreased the PSAP mRNA level. In addition, both the intracellular and extracellular PSAP and saposin C protein levels in the two PSAP-KD clones (P5 and P16 in PC-3 and P15 and P32 in DU-145 cell line) were significantly reduced by ≥70% as compared with two control clones (C1 and C3 in PC-3 and C9 and C13 in DU-145 cell line). Using direct cell counting with haemocytometer and MTS cell proliferation assay, we did not find a significant reduction (≤ 5%) in the PSAP-KD cells growth (data not shown). In addition, we also tested the effect of PSAP down-modulation in soft agar colony formation assay. This study also did not show any significant changes in the size or number of colonies in PSAP-KD clones compared to control transfectants. To evaluate the biological activities of PSAP, we generated a mammalian expression vector expressing the biologically active full-length rhPSAP (see additional files 2, 3, 4, 5). Treatment of both the control and PSAP-KD clones with rhPSAP at 0.1 to 10 nM did not stimulate their growth rate (data not shown). Overall, these results suggest that alterations in the intracellular or extracellular PSAP levels do not affect the anchorage-dependent and -independent growth of PC-3 and DU-145 cells.

### PSAP down-modulation decreases PCa cells adhesion, migration, and invasion

During routine cell culture and trypsinization, we noticed that in both cell lines, the PSAP-KD clones were detached more easily than their control clones or parental cell types. Therefore, we investigated cell adhesion to the major components of the basement membrane such as laminin (LN) and fibronectin (FN). We found that PSAP-KD clones showed a significant reduction of cell adhesion on FN- or LN-coated plates as compared with the control clones (Fig. 1C). In the PC-3 cell line, compared to control transfectants, the adhesion of the PSAP-KD clones on FN and LN was reduced by 78% and 71%, respectively. Likewise, the adhesion of the PSAP-KD clones in the DU-145 cells was decreased by 49% on FN and 69% on LN. We obtained a comparable decrease in cell adhesion for the other extracellular matrix (ECM) proteins such as collagen I or IV in the PSAP-KD clones (data not shown). It is noteworthy that the decreased ability of cell adhesion to ECM proteins was associated with clear morphological changes in PSAP-KD clones as compared with their control counterparts. Control transfectants demonstrated morphological indications of adhesion phenotype such as spreading, membrane protrusion and ruffles, and polarity on all ECM proteins examined. In contrast, PSAP-KD cells appear lower in number and condensed with smaller and either delayed or multi-polar membrane protrusion (Fig. 1D).

**Figure 2:**
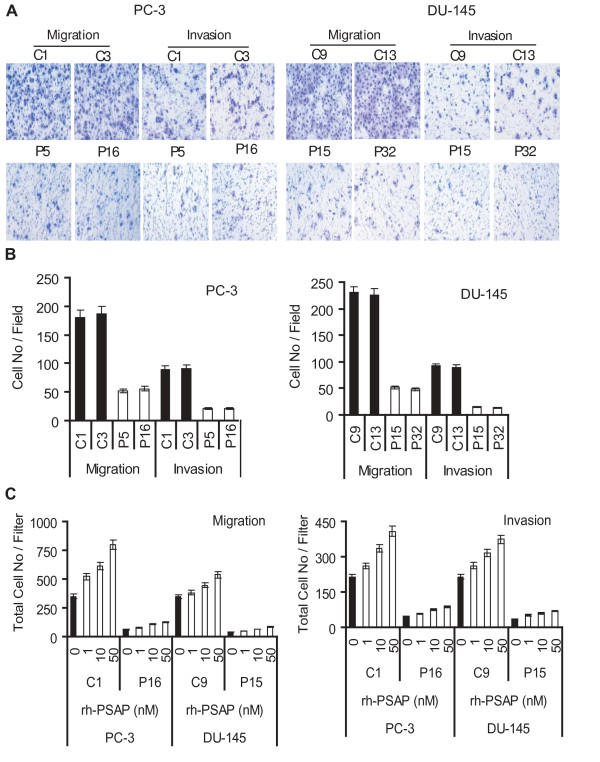
**The PSAP expression correlated with migratory and invasive potential of prostate cancer cell lines**. (A) Transwell filter migration and invasion assays. Stable PSAP-KD clones of PC-3 and DU-145 were seeded on transwell filters and incubated for 24 h. Basal medium containing 5% FBS was used as chemo-attractant. For the invasion assay, the membrane was pre-coated with 50 μg Matrigel. (B) Cells migrated or invaded were counted from ten random fields at 100× magnification using a phase-contrast microscope. (C) Effects of rhPSAP on cell migration and invasion. A representative control and PSAP-KD clone from each cell line was seeded on transwell filters and incubated for 24 h (migration) or 48 h (invasion). As a chemo-attractant, basal medium containing 0.5% FBS and various amount of rhPSAP (0, 1, 10, 50 nM) were included in the lower compartment of the transwell filters. Each bar represented the mean ± SEM of three independent experiments, each in quadruplicates. ANOVA was used to examine the significance of the data (*p *< 0.0001) comparing the PSAP-KD clones relative to control clones in each cell line or among different treatment concentrations for rhPSAP and control.

Defective adhesion might reflect itself in migration and invasion as the two important malignancy-associated phenotypes. Our previous studies revealed that active molecular derivatives of PSAP (i.e. saposin C or TX14A peptide) stimulate PCa cell motility and invasion [14,16]. Next, we examined the effect of PSAP down-modulation on these phenotypes by using the conventional Boyden Chamber (transwell filter) assays. We found that the PSAP-KD clones showed a significant decrease of migration by 70% in PC-3 and 79% in DU-145 compared to the control clones (Fig. 2A &2B). In addition, PSAP down-modulation further reduced the ability of cell invasion through the Matrigel-coated membrane by 78% in PC-3 cells and by 85% in DU-145 cells. We also found that treatment of both control and PSAP-KD cells with rhPSAP in a dose-dependent manner increased their migratory and invasive behavior (Fig. 2C). However, the overall ability of PSAP-KD cells to migrate and invade through Matrigel were significantly less than the control cells indicating a major role for intracellular PSAP expression in the regulation of cell migration and invasion.

### PSAP down-modulation reduces β_1A_-integrin expression

**Figure 3:**
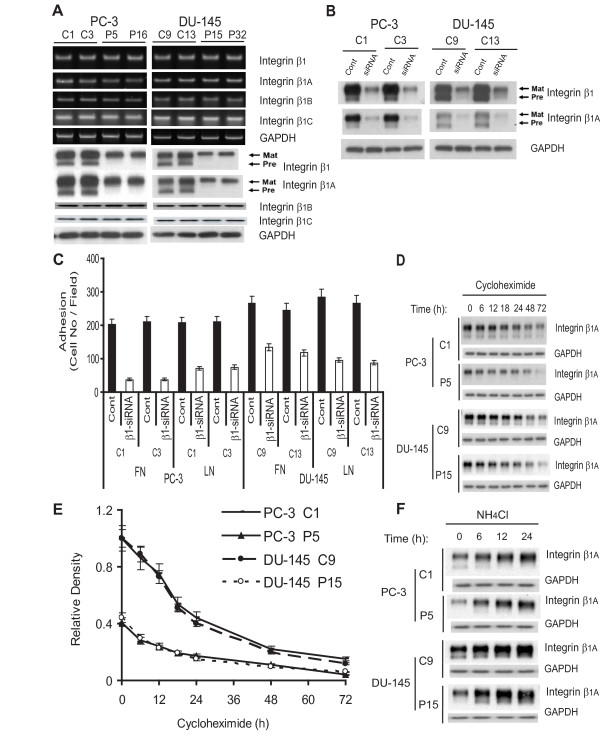
**Effect of PSAP down-modulation on β_1A_-integrin expression and PCa cell adhesion to FN and LN**. (A) PSAP down-modulation reduces β_1_-integrin expression. Total RNA was extracted for RT-PCR with primers specific for integrin-β_1A_, -β_1B_, and -β_1C_, and GAPDH. Cell lysates were analyzed by western blotting with antibodies against β_1_-integrin and its isoforms β_1A_, β_1B_, and β_1C _and GAPDH. (B) Transient down modulation of β_1_-integrin expression. Previously established control stable clones of PC-3 and DU-145 cells were transiently transfected with β_1 _integrin- or scrambled-siRNA oligos. After 48 h, cell lysates were analyzed for integrin β_1 _and β_1A _expression by western blotting. (C) Inhibition of PCa cell adhesion by transient transfection with integrin β_1_-siRNA. After siRNA transfection, cells were subjected to adhesion assay on FN- or LN-coated 96-well plates as described in "Materials and Methods". (D) β_1A_-integrin stability. The steady-state β_1A _protein levels were evaluated by treating cells with cycloheximide (12.5 μg/ml) for up to 72 h and immunoblotting with anti-β_1A _antibody. (E) The half-live of β_1A_-integrin was evaluated by densitometric analysis of immunoblotting bands using the Quantity One software and the β_1A _protein levels were calculated as percentage of non-treatment values after normalization using GAPDH for loading control. (F) Effect of inhibition of lysosomal proteolysis on β_1A_-integrin expression. Cells were incubated in the presence or absence of the lysosomal proteolysis inhibitor, NH_4_Cl (50 mM) for up to 24 h. Cell lysates were analyzed for β_1A _protein expression by immunoblotting. The β_1A_-integrin degradation curve was calculated as described above. *Columns*, mean of three independent samples run together; *bars*, ± SEM, *p *< 0.0001, Two-sample *t*-tests with Satterthwaite corrections were used to compare β_1_-siRNA versus scrambled siRNA oligos transfected cells following adhesion to FN or LN. ANOVA was used to examine the significance of the data (*p *< 0.05) among different cycloheximide treatment periods in PSAP-KD versus control clones. Similar results were obtained from three independent experiments.

Reduction of cell-substrate adhesion in PSAP-KD cells could be the result of changes in the expression and/or usage of adhesion receptors such as the intregrin superfamily which exist as α- and β-subunits. As heterodimers, these subunits could recognize different ECM proteins. Using RT-PCR and immunoblotting, we screened control and PSAP-KD clones of PC-3 and DU-145 cells for α/β-subunit expression. Consistent with previous reports, using specific primers and antibodies against integrin-subunits, we were able to detect moderate to high level of expression for α_1_, α_2_, α_3_, α_5_, α_6_, α_V, _β_3_, and β_4 _integrin subunits [17,18]; no differences between PSAP-KD and control clones were noted. The β_1_-integrin is the most abundant subunit expressed in PCa cells and tissues; it is capable of forming heterodimers that can bind to FN, LN, and collagen IV [19]. Previous studies showed that PCa cells expressed three different β_1 _isoforms: β_1A_, β_1B _and β_1C_, with β_1A _as the most abundant isoform [18]. We found that in PSAP-KD clones only the β_1A _isoform expression at the protein level was reduced while β_1B _or β_1C _did not change. Compared to the control clones, the expression level of both the pre-mature-β_1A _(105 kDa) and the mature β_1A_-isoform (125 kDa) were significantly decreased in PSAP-KD clones (Fig. 3A). As it was expected, the changes in the β_1A _expression pattern were very similar to the total β_1_-integrin. Furthermore, to confirm the role of the β_1A_-integrin expression in PCa cell adhesion on ECM proteins, we repeated the adhesion assays and used control clones that were transiently-transfected with a specific human integrin β_1_-siRNA oligos. The protein levels of total integrin β_1 _as well as β_1A _isoform were reduced by 80-90% in control clones in both cell lines (Fig. 3B). We found that, down-modulation of the β_1_-integrin expression decreased cell adhesion by 83% for FN and 66% for LN in PC-3 and by 52% for FN and 69% for LN in DU-145 (Fig. 3C). These results suggested that reduced expression of β_1A_-integrin expression contributed to the decreased ability of PSAP-KD clones to adhere to basement membrane proteins.

To examine whether changes in protein stability could be responsible for the reduced β_1A _expression in PSAP-KD clones, we investigated the half-life of the β_1A _protein by treating a representative clone from both control and PSAP-KD cells with protein synthesis inhibitor, cycloheximide (CHX). In agreement with previously reported data [20], we found that the β_1A _protein half-life was approximately 20 h in the control clones, while it decreased to ~14 h in the PSAP-KD clones in both cell lines (Fig. 3D &3E). The differences between PSAP-KD and control clones could be due to the enhanced degradation rate of the β_1A _protein in PSAP-KD which allows its earlier disappearance while synthesis of new proteins are inhibited by CHX.

To understand the posttranslational mechanisms responsible for the reduced β_1A _half-life in PSAP-KD cells, we investigated the involvement of the lysosomal-, the calpain- and the ubiquitin-mediated proteolysis pathways. PSAP-KD and control clones were incubated for different time periods (6 to 24 h) with a non-toxic dosage of leupeptin or NH_4_Cl (lysosomal protease inhibitors), ALLN (calpain inhibitor), or MG132 (proteasome inhibitor). Treatment of both the control and PSAP-KD clones, with leupeptin or NH_4_Cl, increased β_1A _expression in a time-dependent manner beginning as early as 6 hours (Fig. 3F). The increase in the β_1A_-integrin expression was more evident in PSAP-KD clones than in the control clones. However, the β_1A _protein expression level was not affected by inhibitors of proteasome or calpain (data not shown). These data show that down-modulation of PSAP via a lysosomal proteolysis-dependent pathway increases β_1A_-integrin degradation rate. Under our experimental conditions, cell viability at the end of the treatment period with CHX or other pharmacological agents was ≥ 95%, as exhibited by a trypan blue dye exclusion assay.

### PSAP down-modulation prevents focal adhesion kinase activation and focal adhesion complex formation

**Figure 4:**
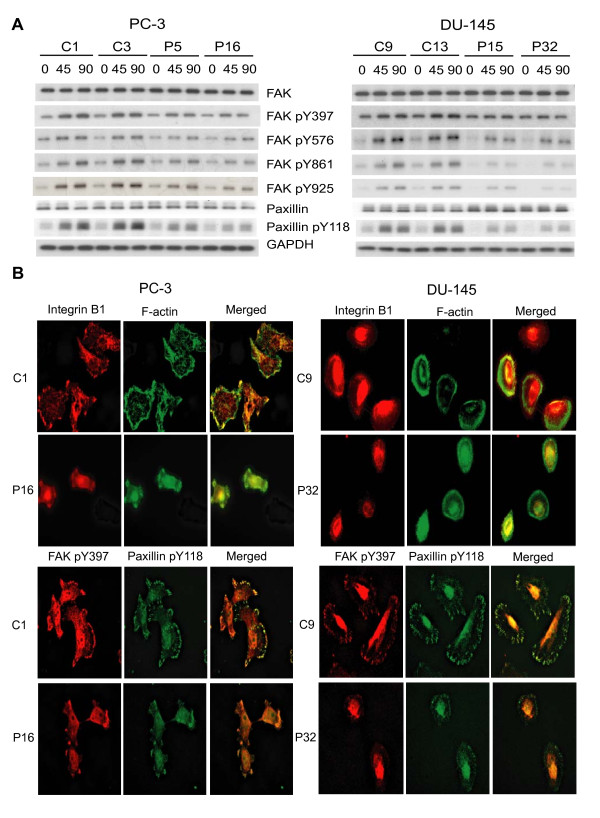
**PSAP down-modulation decreased FAK activity and prevented β_1A_-integrin clustering and proper assembly of focal adhesion complex**. (A) PSAP down-modulation reduced phosphorylation of FAK and paxillin. Cells were incubated in suspension with gentle rotation for 45 min and then plated onto FN- or LN-coated dishes for 45 or 90 min. Whole cell lysates were extracted and equal amount of proteins were used for immunoprecipitation with anti-FAK or-paxillin antibody and immunoblotting with phospho-specific antibodies against Tyr-397, -576, -861, -925 of FAK or Tyr-118 of paxillin. (B) Effect of PSAP down-modulation on β_1A_-integrin clustering and focal adhesion complex assembly. Cells were plated onto FN- or LN-coated slides for 2 h, fixed and permeabilized. Immunofluorescence staining was performed with primary antibodies against integrin β_1A_, FAK pY397 and paxillin pY118 followed by Cy3 (red) or FITC (green)-conjugated secondary antibodies. F-actin was stained by Oregon Green 488-phalloidin (green). All images were taken by a Leica DM RA2 fluorescence microscope. Consistent data were obtained from three independent experiments.

PSAP-KD cells appeared small and condensed and did not show morphological evidence of adhesion phenotype such as spreading, directional membrane protrusion, and ruffles. These data promoted us to investigate the activity, expression, or subcellular localization of certain structural molecules (actin and vinculin), focal adhesion kinase (FAK) as the most important integrin-regulated signaling molecule, and adaptor protein (i.e., paxillin) which are collectively involved in the assembly of focal adhesion complex. Using whole cell lysates prepared from subconfluent cells and following their adhesion to FN or LN, we examined the phosphorylation of FAK at different tyrosine residues and paxillin by immunoprecipitation (IP) of FAK and western blotting with phospho-specific antibodies. As shown in Fig. 4A, total FAK and paxillin protein levels were not affected by PSAP down-modulation. FAK was constitutively phosphorylated on tyrosine residues in control transfectants to the levels similar to PSAP-KD clones. Following 45 or 90 min adhesion to FN or LN, FAK phosphorylation at Tyr-397, Tyr-576, Tyr-861, and Tyr-925 and the level of paxillin phosphorylation at Tyr-118 increased at higher amounts in the control clones than the PSAP-KD clones (Fig. 4A). To visualize the impairment of cell adhesion in relation to the changes in β_1A_-integrin and the assembly of focal adhesion plaque, we used immunofluoresence (IF) staining of a representative clone of the control and PSAP-KD cells. As shown in Fig. 4B, the control cells spread out on the ECM-coated slides and showed a strong β_1_-integrin staining that was mainly localized at or near the cell membrane region, suggesting a functionally activated β_1_-integrin. However, the PSAP-KD cells showed a small and round morphology and a weak β_1_-integrin staining which remained non-clustered and largely in the cytoplasmic region. Furthermore, the control cells formed several focal contacts as visualized by phospho-specific antibodies against FAK (Tyr-397) and paxillin (Tyr-118). The control cells also exhibited a greater extent of co-localization of FAK and paxillin proteins. However, the PSAP-KD cells showed clearly attenuated activation of focal adhesions characterized by a smaller size and lesse number of focal contacts as well as irregular localization of FAK and paxillin (Fig. 4B). By using the antibody against vinculin, another cytoskeletal protein, similar attenuation in the formation of focal adhesions was also observed in the PSAP-KD clones (data not shown).

In addition, stress fibers (F-actin) were also arranged as long fibers co-localized with vinculin and in parallel with membrane protrusions in control transfectants. In contrast, such topological evidence of adhesion phenotype was absent in PSAP-KD cells. Overall, these data suggest that the reduction of β_1A_-integrin expression secondary to PSAP-down modulation via the interruption of the "inside-out" signaling mechanism significantly inhibits FAK activity and the proper assembly of focal adhesion complex and contributes to impaired cell adhesion and migratory phenotype in PSAP-KD cells.

### PSAP down-modulation decreases cathepsin D expression and proteolytic activity in PCa cells

**Figure 5:**
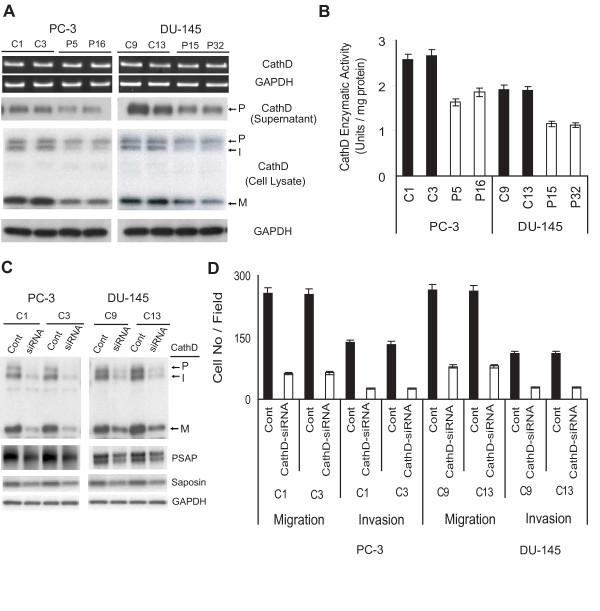
**Down-regulation of cathepsin D expression and activity decreased migration and invasion in PSAP-KD cells**. (A) PSAP down-modulation reduced CathD expression and activity. Total RNA was subjected to RT-PCR using specific primers for CathD. Cell lysates and culture supernatants were prepared from parallel dishes and analyzed by immunoblotting with an anti-CathD monoclonal antibody which recognizes proCathD (P), intermediate CathD (I), and mature CathD (M). (B) Whole cell extracts were also assayed for CathD enzymatic activity using a kit with a fluorimetric substrate. The enzymatic activity of CathD was calculated as units/mg total protein. *Columns*, mean of three independent samples run together; *bars*, ± SEM. ANOVA was used to examine the significance of the data (*p *< 0.0001) comparing PSAP-KD clones versus control clones. (C) Transient down-modulation of CathD and its effect on PSAP expression. Control clones of PC-3 and DU-145 cell lines were transiently transfected with specific CathD- or control-siRNA oligos. After 48 h, cell lysates were analyzed for CathD, PSAP, and saposin C expression by immunoblotting. (D) Migration and invasion assays were performed on parallel-transfected tissue culture plates as described in the legend to Fig. 2. *Columns*, mean of three independent samples run together; *bars*, ± SEM, *p *< 0.0001, Two-sample *t*-tests with Satterthwaite corrections were used to compare CathD-siRNA versus scrambled-siRNA oligos transfected cells. Similar results were obtained from three independent experiments.

The multi-step process of invasion phenotype requires the involvement of matrix-degrading proteolytic enzymes. Among different classes of proteolytic enzymes, several lines of evidence demonstrated a dynamic active physical and functional interaction between CathD and PSAP [4,6,21]. Therefore, we examined if down-modulation of PSAP affects CathD expression and activity. As shown in Fig. 5A, CathD mRNA expression was not affected by PSAP down-modulation in any of the cell lines investigated. However, we observed a significant reduction in the expression levels of inactive proCathD (~53 KDa), active intermediate (~48 KDa), and mature (~31 KDa) forms in all PSAP-KD clones compared to their control counterparts. The secreted levels of proCathD were also reduced by PSAP down-modulation. In addition, our analysis showed that CathD proteolytic activity in the PSAP-KD clones decreased by 22% in PC-3 cells and by 48% in DU-145 cells (Fig. 5B).

To assess the involvement of CathD in PCa cell invasion, the PC-3 and DU-145 control clones were transiently transfected with human CathD siRNA-oligos. As shown in Fig. 5C, CathD-siRNA reduced CathD expression by 90% in both cell lines. Interestingly, knock-down of CathD expression also decreased the intracellular expression of PSAP and saposin C (Fig. 5C). In addition, in vitro migration and invasion assays revealed that CathD-siRNA decreased cell migration by 76% in PC-3 and by 71% in DU-145, as well as cell invasion by 82% in PC-3 and 77% in DU-145 (Fig. 5D). Cell viability assays showed that the decrease of cell migration and invasion was independent of cell proliferation. Overall, these data strongly support a close metabolic and functional relationship between PSAP and ProCathD in the process of migration and invasion in PCa cells.

### PSAP down-modulation increases ceramide levels in PCa cells

**Figure 6:**
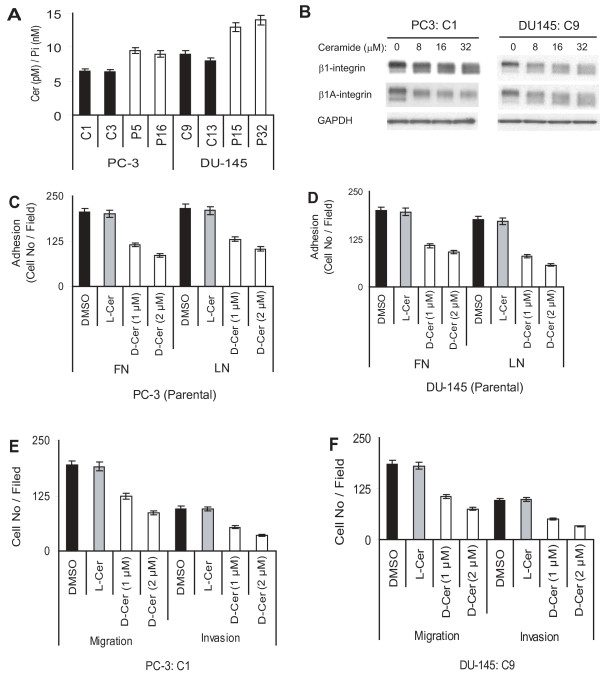
**Effect of ceramide on PCa cell adhesion, migration, and invasion**. (A) PSAP down-modulation increased Cer levels in PSAP-KD cells. PSAP-KD and control clones of PC-3 and DU-145 cells were subjected to matrix-assisted laser desorption mass spectrometric analysis as described in "Materials and Methods". The assay was performed in duplicate and repeated twice independently. Cer content was quantified and calibrated to the intracellular phosphate (Pi) level and depicted as Cer (pM)/Pi (nM). (B) Effect of Cer on β_1A_-integrin expression. Control clones of PC-3 and DU-145 cells were treated with active or inactive Cer analogs or vehicle (DMSO) at the indicated concentrations for 36 h. Cell lysates were subjected to immunoblotting with specific antibodies against total β_1_-integrin or β_1A_-isoform. Inactive Cer analog and DMSO did not affect integrins expression level. GAPDH antibody was used as control loading. (C and D) Effect of Cer on PCa cell adhesion. Parental PC-3 and DU-145 cells were treated with cell permeable natural Cer analog (C6-*D-e*-Cer; D-Cer), inactive Cer (C6-*L-e*-Cer; L-Cer), or vehicle (DMSO) at 1 or 2 μM for three to five days and then, subjected to cell adhesion on FN- or LN-coated plates as described in the legend to Fig. 1. (E and F) Effect of Cer on PCa cell migration and invasion. The effect of Cer on migration and invasion of control PC-3 and DU-145 transfectants was examined by treating cells with active or inactive Cer analogs (described above) followed by migration and invasion assay as explained in the legend to Fig. 2. Each bar represents the mean ± SEM of three independent experiments. ANOVA models with Dunnett and Tukey corrections were used to compare cells treated with DMSO, L-Cer, or D-Cer. Statistically significant differences were set at *p *< 0.05. Consistent data were obtained from three independent experiments.

PSAP is the precursor of saposins which serve as the essential co-factors of lysosomal sphingolipid hydrolases. Therefore, the presence and relative abundance of PSAP greatly influence the balance between sphingolipid synthesis and degradation. In the lysosomes, saposins, derived from PSAP, degrade Cer which physiologically serves as one of the most important second messenger systems in the regulation of fundamental biological processes such as growth, differentiation, cell adhesion, and migration as well as the activation of several important signaling pathways. By using MALDI-mass spectrometry analysis, we found that a number of cellular Cer with various hydro-carbon lengths, including Cer-C16, -C18, and -C24 are accumulated in the PSAP-KD clones. Total Cer levels in PSAP-KD clones were increased by 46% and 56% in PC-3 and DU-145 cells, respectively (Fig. 6A). However, we did not observe a significant change in the levels of sphingosine, a metabolic product of Cer, as well as glycosphingolipids with short oligosaccharides, such as lactosyl-Cer and glucosyl-Cer (data not shown). Next, we examined if Cer can affect β_1A_-integrin expression in stable control clones of PC-3 and DU-145 cells (Fig. 6B). C6-*D*-*e*-Cer treatment decreased both the expression and maturation of β_1A_-integrin. Inactive Cer failed to induce any changes in β_1A_-integrin expression (data not shown). To determine whether intracellular Cer accumulation might be responsible for the impairment of cell adhesion to ECM proteins, we investigated the possible effects of Cer on cell adhesion, migration and invasion by treating the cells with C6-*D*-*e*-Cer. C6-*D*-*e*-Cer in a dose-dependent manner decreased cell adhesion to FN and LN by ≥ 50% in control clones from both cell lines (Fig. 6C &6D). It is noteworthy that the inhibition of cell adhesion on FN and LN was associated with clear morphological features such as decreased spreading of the cells and lack of polarity and membrane protrusions (data not shown) similar to our observation in Fig. 1D and 4B. In addition, C6-*D-e*-Cer, in a dose-dependent manner decreased cell migration and invasion by 56% and 64% in control clones of PC-3 cells and by 59% and 66% in control clones of DU-145 cells, respectively (Fig. 6E &6F). However, neither the morphological alterations nor inhibition of cell adhesion, migration, and invasion were induced by inactive Cer analog C6-*L-e*-Cer or the C6-*D-t*-Cer, the unnatural stereoisomeres of C6-*D-e*-Cer, which suggested that these phenotypes are highly dependent on native Cer configuration. Treatment of cells with 8 to 32 μM of C6-*D*-*e*-Cer or its inactive enantiomers C6-*L*-*e*-Cer and C6-*D*-*t*-Cer for 24 or 36 h followed by a trypan blue exclusion assay showed ≥ 95% cell viability.

## Discussion

We employed an RNA-interference strategy to investigate the molecular events underlying PSAP regulation of metastatic PCa cell invasion. Stable down-modulation of PSAP decreased CathD expression and proteolytic activity, migration, and invasion of the highly invasive and metastatic PCa cells. In vitro and in vivo studies have implicated a role for CathD in PCa growth, invasion, and metastasis [22-25]. Data from transient transfection studies presented here further support a relationship between ProCathD and PSAP. The two precursors PSAP and proCathD are glycoproteins that originate in the endoplasmic reticulum (ER) which travel together as a PSAP-proCathD complex and reach the lysosomes by intracellular trafficking [4,21,26,27]. In endosomal and/or lysosomal compartments, they undergo mutual proteolytic processing to become the final mature products, saposins and CathD. PSAP accelerates the activation of proCathD and stimulates its autocatalytic activity, generating the enzymatically active intermediate and mature CathD. In turn, CathD catalyzes the production of saposins from PSAP [4,27]. In our study, most of the observed decrease in intracellular CathD was in the enzymatically active forms (intermediate and mature) rather than in proCathD levels. This data suggests that PSAP down-modulation not only affect proCathD conversion to CathD, but might also influence CathD stability and/or synthesis. Taken together our data suggest a cooperative interaction between PSAP and CathD in PCa cell migration and invasion.

In PCa cells, β_1_-integrin is the most abundant and ubiquitously expressed subunit [19]. Experimental modifications of β_1_-integrin have been demonstratred to affect development, cell proliferation, migration, and activation of downstream FAK-Src signaling [28,29]. We discovered that, β_1A_-integrin isoform is not only the most abundant isoform, but also that down-modulating cellular PSAP levels significantly reduced its expression in the highly invasive and metastatic PCa cell lines, PC-3, DU-145, and C4-2B (data not shown).

Several studies have demonstrated that, upon engagement with ECM components, integrins reorganize to form focal adhesion complexes, activate FAK autophosphorylation at Tyr-397, and establish a mechanical linkage with cytoskeletal molecules such as actin and vinculin, which control cell shape and motility [30]. FAK phosphorylation at Tyr-397 also creates a high-binding affinity site for Src-homology 2 (SH2) domain of Src-family kinases and establishes FAK-Src signaling complex. This association leads to the Src transphosphorylation of FAK within its kinase domain of the activation loop (Tyr-576) and its C-terminal domain (Tyr-861 and -925) and to the activation of downstream adaptor molecules such as paxillin, by phosphorylation at Tyr-118 [31]. Consistent with the role of integrins in FAK-Src signaling regulation and downstream activation of adaptor molecules, we found that decreasing β_1A_-integrin expression disrupted these processes in several ways: a) loss of directional membrane protrusion and ruffles and clustering of β_1_-integrin and FAK, b) inability to form focal adhesion complex, c) decreased Src-binding to FAK (see additional file 6), d) significant reduction of phosphorylative activity of FAK at Tyr-397, -576, -861, and -925, and e) decreased phosphorylation of paxillin at Tyr-118 in PSAP-KD cells. These data provide a classical example whereby interruption of integrin-regulated FAK-Src signaling secondary to down-modulation of PSAP (as a lysosomal protein) leads to a less adhesive and motile phenotype in PCa cells.

The key findings of this report are the significant reduction of Src binding to FAK and the lack of proper assembly of focal adhesion complex in PSAP knockdown cells. Together, they highlight the importance of PSAP and saposin C in regulating "inside-out" integrin-mediated signal transduction pathway(s) leading to decreased PCa cell migration and invasion. Based on our data, it appears the observed structural and functional outcomes occur primarily due to reduced β_1A_-integrin expression following PSAP down modulation. In addition, reduction of Src binding to FAK was paralleled with decreased Src activity (phosphorylation) in PSAP-KD cells (data not shown) and did not affect the activity level of its upstream targets MAPK and PI3K/Akt (data not shown). As natural cell membrane and intracellular proteins, PSAP and its active molecular derivatives, saposin C and its neuro-active domain (prosaptide), might also interact with Src alone or in association with focal adhesion complex and other interactive adaptor proteins (e.g., paxillin) to stabilize the dynamic state of focal adhesion plaques.

Accumulated Cer levels secondary to PSAP down-modulation which lead inevitably to reduction of saposins may be responsible for decreased β_1A _expression. In support of this assertion, we found that exogenous Cer not only decreased PCa cell adhesion, migration, and invasion, but also reduced β_1A_-integrin expression in control clones of PC-3 and DU-145 cell lines. It has been reported that Cer could inhibit integrin β_1_-glycosylation and -trafficking to cell surface by disrupting the function of Golgi complexes [32]. We observed that PSAP down-modulation induced the accumulation of cellular Cer without affecting the levels of glycosphingolipids. This result is somewhat different from those other studies of total PASP deficiency in patients and in experimental mouse models, in which significant accumulation of Cer as well as lactosyl-Cer and glucosyl-Cer has been observed [9,10,33]. We speculate that the balance of Cer metabolism is more sensitive to the relative changes in PSAP expression than is the metabolism of glycosphingolipids, which essentially dependes on the presence of a low (threshold) PSAP level, similar to the residual amount of PSAP in the PSAP-KD clones, which is comparable to normal prostate epithelial cells (data not shown). It is noteworthy that the endogenous Cer levels are coordinately regulated by several specialized enzymes and hydrolases which produce Cer or use Cer as substrate [34]. Elevated PSAP expression may shift the balance of Cer by activating certain hydrolases or even by directly regulating their expression through functional saposins. For example, saposin D can stimulate the activity of acid ceramidase, which mediates the conversion of Cer into sphingosine [35]. This hypothesis is supported by our finding that ceramidase expression is reduced in PSAP-KD cells (unpublished data). The Cer level is frequently decreased in cancer cells and correlates inversely with the degree of malignant progression [36]. Therefore, it is conceivable that PSAP overexpression may greatly contributes to Cer-level reduction in invasive and metastatic cancer cells. Considering the complexity of Cer as a bioactive sphinogolipid, the underlying mechanisms by which Cer inhibits PCa cell motility and invasiveness require further detailed investigation.

Our data indicate a role for soluble PSAP as a paracrine regulatory factor in migration and invasion. Based on our study, this paracrine regulatory effect is not sufficient to bypass the intracellular regulatory mechanisms responsible for significant suppression of migratory and invasive phenotypes secondary to PSAP down-modulation. It is likely that the receptor-mediated signaling mechanisms and post-receptor downstream effectors responsible for the paracrine effect of PSAP may be different from the intracrine regulatory pathways.

Our previous studies also showed that exogenous saposin C and prosaptide treatment could stimulate PCa cell growth, involving activating several signaling pathways [14]. However, our current data show that under our experimental conditions, the growth properties of PCa cells was not affected by either intracellular down-modulation of PSAP or treatment with rhPSAP. Furthermore, neither PSAP down-modulation nor rhPSAP treatment affected the MAPK and PI3K activity level (unpublished data). Therefore, the observed effect of exogenous saposin C does not necessarily reflect the physiological function of extracellularly secreted PSAP or an intracellular pool of this protein.

PSAP has been demonstrated to be overexpressed in conditioned media of estrogen receptor (ER)-positive MCF-7 and ER-negative MDA-MB-231 breast cancer cell lines as well as in a human SV40-transformed breast epithelial cells, HBL100 [37]. In MCF-7 conditioned media, the PSAP expression pattern closely resembled that of proCathD. Interestingly, the same authors demonstrated that estrogen (17β-estradiol) increased secretion of both proteins in a dose-dependent manner. These observations together with our data support the hypothesis that the close functional association between proCathD and PSAP may eliminate tissue barriers by facilitating proteolytic degradation of basement membrane glycoproteins. PSAP was also identified as a gene with causative role during functional screening for tamoxifen-resistance in breast cancer cell line, ZR-75-1 [28]. Further investigation of clinical samples using qRT-PCR analysis of mRNA levels in 223 ER-positive primary breast cancers from patients who had recurrent metastatic disease and were treated with tamoxifen as a first-line therapy, revealed a high PSAP expression level for 182 out of 223 patients. In addition, Cox univariate and multivariate analyses for progression-free survival correlated the high PSAP expression levels in these patients with shorter progression-free survival [29]. Independently, using Mass Spectrometry based-proteomic analyses and qRT-PCR for comparative analysis of non-metastatic primary breast cancer and lymph node metastases, PSAP was found to be significantly increased (2 fold) in lymph node metastasis [38]. Similarly, in our previous studies using punch biopsy samples of metastatic PCa, genomic amplification of PSAP was detected in 2 out of 5 lymph node metastases [13]. In addition to breast cancer and PCa, in a comparative analysis of the secretomes of an immortalized pancreatic duct normal epithelial cell (HPDE) and a pancreatic ductal adenocarcinoma cell line (Panc1), PSAP expression in Panc1 was found to 11-fold higher than in the HPDE cell line [39]. Interestingly, PSAP upregulation in Panc1 was associated with CathD (6.7-fold) and β_1_-integrin (3.4-fold) overexpression. While these reports suggest a role for PSAP in invasive and metastatic progression of prostate, breast, and pancreatic tumors, a recent report has suggested that PSAP may inhibit breast and PCa metastasis by paracrine and endocrine stimulation of thrombospondin-1 expression in a p53-dependent manner in fibroblasts of primary tumors and distant metastases [40]. With respect to PCa, the study was based on PC-3M, a metastatic subline of PC-3 cell line. In addition, the authors used PC-3M-LN4, a lymph node-metastatic subline of PC-3M that had been subjected to four cycles of injection to prostate and harvesting from the lymph node of athymic nude mice [41]. Due to extensive clonal selection, it is difficult, if not impossible, to differentiate between the influence of clonal selection and a cause-and-effect relationship for the PSAP contribution as an inhibitor of PCa metastasis. Our analysis of three independent PCa progression models based on isogenic cell lines has revealed a steady-state increase in PSAP expression levels in invasive and metastatic cells as compared to their parental cells (see additional file 1). These data show that PSAP expression in PC-3M is at least 3-fold higher than in its isogenic parental cell line, PC-3, indicating that, upon metastatic progression, PSAP expression increases. By analyzing gene microarray expression data from different sources, the authors also reported that the relative PSAP mRNA expression in metastatic PCa was 30% lower than in localized primary tumors [40]. This analysis is based solely on bioinformatics evaluation which does not necessarily represent the mRNA and protein expression levels of tumor cells. As such, a cause-and-effect relationship between PSAP and the complex multistep process of metastatic phenotype in PCa can not be concluded from the study. Clarification of PSAP's role in invasive and metastatic progression of PCa and other malignancies requires additional detailed investigations.

In summary, we provide mechanistic evidence that PSAP down-modulation upregulates Cer levels, decreases β_1A_-integrin and CathD expression, attenuates the "inside-out" integrin-signaling pathway, and significantly decreases PCa cell adhesion, migration, and invasion. The fact that PSAP is frequently overexpressed in human malignant cells warrants further investigation of its role in carcinogenesis and in invasive and metastatic progression of cancer cells.

## Materials and methods

### Cell culture

Cell lines used in this study were essentially maintained as described before [13,14]. Cycloheximide (CHX), leupeptin, MG-132, and ALLN were obtained from Sigma (Saint Louis, MO).

### Expression and purification of recombinant human PSAP in CHO-K1 cells

The full-length cDNA of *PSAP *gene (GenBank Accession No. NP002769) was synthesized, tagged at the C-terminal with hexa-histidine (His_6_), and subcloned into the mammalian expression vector pSectag2A (Invitrogen, Carlsbad, CA). The pSectag2A vector contained the Igκ leader sequence which allows the secretion of recombinant proteins. After bacterial transformation, the sequence accuracy was verified by automated sequencing in both directions. Stable CHO-K1 clones expressing high levels of the secreted recombinant human (rh)-PSAP was obtained using Zeocin as a selection antibiotic. Recombinant PSAP protein was purified from culture supernatant using imidazole and Ni-NTA Superflow Resins (Qiagen, Santa Clara, CA). The molecular size of recombinant PSAP expressed in CHO-K1 cells was similar to that of native PSAP secreted by PC-3 cells. The size and purity of the purified proteins were determined by using 4-20% Tris-Glycine gel electrophoresis, coomassie blue staining, silver staining, and western blotting with previously characterized anti-PSAP antibodies (see additional files 2, 3, 4, 5) [14].

### Establishment of stable transfectants of PSAP knock-down cell lines

Cells were seeded at 2 × 10^5 ^per well in 6-well plates overnight and transfected with 2 μg short hairpin (sh)-RNA plasmid containing a siRNA sequence targeted against human PSAP or a scrambled control sequence (SuperArray Biosciences, Germantown, MD) and 5 μl Lipofectamine 2000 according to the manufacturer's instructions (Invitrogen, Carlsbad, CA). After 8 hours of incubation at 37°C, the transfection medium was removed and cells were cultured in complete medium for 48 h. Cells were trypsinized and cultured in the presence of 1 mg/ml G418 for the selection of antibiotic-resistant colonies over a period of 2 to 3 weeks. Several PSAP-knockdown (KD) and -control clones were isolated and analyzed for PSAP expression by western blotting and RT-PCR. We randomly selected two PSAP-KD clones (P4 and P7 for PC-3 cell line and P15 and P32 for DU-145) and two control clones (C4 and C8 for PC-3 cell line and C9 and C13 for DU-145 cell line) for functional studies. The stable cell lines were routinely examined for PSAP expression and maintained in the complete medium containing 300 μg/ml of G418.

### Transient transfection assays

Cells were seeded in 6-well plates overnight and transfected with 50 pmol of human CathD-, integrin-β_1_, or control-siRNA oligos (Santa Cruz Biotechnology, Santa Cruz, CA) and 5 μl Lipofectamine RNAiMAX for 8 h. The transfected cells were cultured in complete medium for 16 h and then, in basal medium for additional 24 h before performing functional assays or harvesting cell lysates and/or supernatants for protein expression analyses.

### RNA extraction, cDNA synthesis, and semi-quantitative RT-PCR

RNA was isolated by using the RNeasy Kit according to the manufacturer's instructions (Qiagen, Santa Clara, CA). For cDNAsynthesis, the template (5 μg RNA per sample) was reverse-transcribed using AffinityScript cDNA Synthesis Kit (Stratagene, La Jolla, CA). Semi-quantitative PCR was performed in total 20 μl volume containing 1 μl cDNA, 0.2 μM dNTPs, 0.4 μM primers, and 0.4 μl Taq DNA polymerase (Promega, Madison, MI). Primers were synthesized by Integrated DNA Technologies Inc (San Jose, CA). The oligonucleotides used (according to the human gene sequences with accession numbers: NM_002211 for β_1A_, NM_033666 for β_1B_, NM_033667 for β_1C_, NM_002778 for PSAP, NM_001909 for CathD, and NM_002046 for GAPDH, as deposited at the NCBI/genome data bank) were as follows: β_1A_, 5'-AGAATCCAGAGTGTCCCACTGG-3' (sense) and 5'-TTTCCCTCATACTTC GGATTG-3' (antisense); β_1B_, 5'-AAGACTTATGTATTAGCTGTCAG-3' (sense) and 5'-CCATTGAATAGCTTG CTACAC-3' (antisense); β_1C_, 5'-TCTGTCGCCCAGCCTGGAGTG-3' (sense) and 5'-TTTCCCTCATACTTCGGATTG-3' (antisense); PSAP, 5'- CCA GAG CTG GAC ATG ACT GA-3' (sense) and 5'-CAGTTCCCAACAAGGGCTTA-3' (antisense); CathD, 5'-CTGCACAAGTTCACGTCCAT-3' (sense) and 5'-TTCTGCTGCATCAGGTTGTC-3' (antisense); and GAPDH, 5'-GGTCGGAGTCAACGGATTTGGTCG-3' (sense) and 5'-CCTCCGACGCCTGCTTCACCAC-3' (antisense). After cDNA synthesis, PCR was completed using a T-gradient model (Biometra, Horsham, PA) under the following conditions: a denaturation cycle at 95°C for 2 min, 95°C for 45 s, annealing at 58°C for 45 s and elongation at 72°C for 40 s, and a final extension at 72°C for 5 min. The sizes of amplified cDNA fragments and the number of PCR cycles were: 238 bp/20 cycles for β_1A_, 278 bp/32 cycles for β_1B_, 172 bp/32 cycles for β_1C_, 1000 bp/20 cycles for PSAP, 590 bp/20 cycles for CathD, and 780 bp/20 cycles for GAPDH. The PCR product was confirmed as a single band using 1.5% agarose gel electrophoresis. A non-template control was included in each PCR experiment. Each experiment was repeated three times independently.

### Protein extraction, immunoblotting, and immunoprecipitation

Cell-free culture supernatants were collected and concentrated up to 10 times by using a centrifuge concentrator with a 3.0 kDa molecular weight cut-off membrane (Vivascience, Stonehouse, UK). Protein samples (2 μg for CathD and 5 μg for PSAP) were subjected to SDS-PAGE and immunoblotting as previously described [24]. Normalization of culture supernatants was based on the total cell number and/or protein content. Whole cell lysates were also prepared from the same tissue culture plates and used for immunoblotting (2 μg for CathD and 15 μg for PSAP). Membranes were probed with mouse anti-human PSAP (1 μg/ml; Abnova, Taibei, Taiwan) or mouse anti-human CathD (clone CTD-19 at 1:2000; Santa Cruz) antibodies and signals were detected by ECL detection system (GE Healthcare, London, UK). Direct immunoblotting for cell adhesion molecules was performed on protein lysates: 1 μg for integrin β_1_,2 μg for integrin β_1A_, 20 μg for integrin β_1B_, 30 μg for integrin β_1C_, and 5 μg for paxillin. We used the following antibodies for immunoblotting of cell adhesion molecules: Mouse anti-integrin β_1 _(Santa Cruz, clone JB1B at 1:200), rabbit anti-integrin β_1A _(Millipore, AB1952P at 1:1000), rabbit anti-integrin β_1B _and β_1C _antibodies provided by Dr. L.R. Languino, University of Massachusetts (at 1:100), monoclonal anti-paxillin (Millipore, clone 5H11, 1:1000), and rabbit anti-paxillin-pY118 (Cell signaling, 1:1000). Anti-GAPDH (Santa Cruz; 1:4000) was used as control loading.

For immunoprecipitation of cell adhesion molecules, tissue culture plates were coated with 10 μg/ml fibronectin (FN, Sigma, Saint Louis, MO) or laminin-1 (LN, R&D Systems, Minnneapolis, MN) overnight at 4°C and blocked with 1% BSA/PBS for 1 h at room temperature. Subconfluent cultured cells were washed with PBS, incubated with Versene (Invitrogen) for 30 min at 37°C and a final incubation with 0.0025% trypsin for 10 min. The detached cells were collected by centrifugation and 6 × 10^6 ^cells in basal medium and were incubated for 45 min at 37°C with gentle rotation. Equal volume of cell suspension were either lysed immediately or added to FN- or LN-coated plates for 45 or 90 min. At the end of incubation periods, plates were washed with cold PBS on ice and cells were collected by centrifugation at 300 × g for 3 min. One mg protein lysates were incubated with 1 μg anti-FAK monoclonal antibody (clone 4.47, Millipore, Billerica, MA) overnight followed by 2 h incubation with rotation at 4°C in the presence of 20 μl Protein A/G agarose beads (Santa Cruz). After three washes with the lysis buffer, the beads were re-suspended in sample-loading buffer and aliquots of cleared supernatant were subjected to SDS-PAGE and immunoblotting with a mouse anti-FAK (clone 4.47; Millipore) at 1:1000 dilution, anti-FAK-pY397 (MAB1144; Millipore) at 1:250 dilution, rabbit anti-FAK-pY576 (Invitrogen) at 1:1000 dilution, anti-FAK-pY861 (Invitrogen) at 1:1000 dilution, or rabbit anti-FAK-pY925 (Cell Signaling) at 1:1000 dilution. HRP-conjugated secondary antibodies (Santa Cruz) were used at 1:1000 dilutions. Where indicated, subconfluent culture plates were incubated in the basal medium for 24 h and then, incubated with the protein synthesis inhibitor, CHX (12.5 μg/ml; Sigma), the lysosomal protease inhibitors, leupeptin (100 μM; Sigma) or NH4Cl (50 mM), the calpain inhibitor ALLN (10 μM; Sigma), the proteasome inhibitor MG132 (10 μM; Sigma), or the vehicle alone (DMSO; Sigma) before harvesting protein lysates. Immunoblotting bands were quantified by densitometric analysis using the Quantity One software (Bio-Rad) and the protein levels were calculated as percentage of non-treated cells with GAPDH normalization.

### Cell proliferation assays

To evaluate the effect of PSAP down-modulation on cell proliferation, PSAP-KD and control clones were seeded at 2 × 10^5 ^in 10 cm tissue culture dishes in complete medium and incubated for 2, 4, or 6 days. At the end of incubation period, cells were harvested by trypsinization and viable cell number was determined by trypan blue exclusion assay using a hematocytometer. To determine the effect of rhPSAP on cell growth, 2 × 10^3 ^cells per well were seeded in 96-well plates in complete medium for 2 days and, after washing the plates with PBS, cells were incubated in the presence or absence of rhPSAP at 0.1, 1, 10 nM or 0.5% FBS in basal medium containing 0.1% BSA. After 2 days, the cell number was measured by MTS assay using CellTiter 96 AQueous One Solution Cell Proliferation/Cytotoxicity Assay Kit according to manufacturer's instructions (Promega). Briefly, 20 μl MTS solution was added to each well for 2 h incubation and the absorbance at 490 nm was determined. We used twelve replicates for each treatment condition.

### Cell adhesion assays

To determine the effect of PSAP down-modulation on adhesion, subconfluent cultured cells were harvested by versene treatment as described in the immunoprecipitation assays for cell adhesion molecules and seeded at 1.5 × 10^4 ^cells/well in basal medium on FN- or LN-coated 96-well plates as described above. After 2 h of incubation at 37°C, cells were washed twice with PBS, fixed with 10% formaldehyde, and stained with 0.25% tolouidine blue each for 15 min at room temperature. Images were taken at 100× magnification by a video camera (Nikon DU-S1) fitted to a microscope. The adhered cells were counted from ten randomly chosen fields in at least six independent wells. The experiment was repeated three times independently.

### Cell migration and invasion assays

The effect of PSAP down-modulation on cell migration and invasion was performed using 8-μm transwell filters (Costar, Corning, NY) with modification as described previously [14]. For the invasion assay, the upper compartment was coated with 50 μg Matrigel (BD Biosciences, San Jose, CA) to form a matrix barrier. A suspension of cells (5 × 10^4 ^for PC-3 or 2 × 10^4 ^for DU-145) in basal medium containing 0.1% BSA was added to the upper compartment. The lower compartment was filled with 400 μl basal medium containing 5% FBS as chemoattractant. After 48 h for PC-3 or 24 h for DU-145, the non-migratory cells on the upper surface were removed by a cotton swab and the cells on the lower surface were fixed and stained with the Diff-Quick solution (Dade Behring, Deerfield, Illinois). To test the effect of rhPSAP on cell migration and invasion in stable transfectants, 2 × 10^4 ^PC-3 or 1 × 10^4 ^DU-145 cells were added to each well and incubated 24 h for migration or 48 h for invasion. Basal medium containing 0.5% FBS in the absence or presence of rhPSAP at 0.1, 1, 10, or 50 nM was used as chemoattractant in the lower transwell compartment. Migrated or invaded cells in each transwell filter were counted. Imaging and cell counting were performed as described for cell adhesion assays. The experiment was performed in quadruplicates and repeated at least three times independently.

## Cathepsin D activity assays

Cells were grown up to 70% confluency in their maintenance medium and serum starved for 24 h. Cells were collected in extraction buffer containing 10 mM HEPES pH 7.0, 10 mM KCl, 1.5 mM MgCl_2_, 0.5% CHAPS and subjected to three freeze-thaw cycles. After centrifugation at 300 × g for 10 min, the clarified crude cell extracts were subjected to CathD Assay Kit (Sigma) based on the hydrolysis by the enzyme of an internally quenched fluorimetric substrate (i.e., MCA) according to manufacturer's instruction.

## Immunofluorescence staining and microscopic analysis

To visualize the effect of PSAP down-modulation on cell adhesion molecules, subconfluent culture plates were detached by versene treatment as described for the immunoprecipitation assays of cell adhesion molecules. Cell suspensions were incubated in a basal medium for 45 min at 37°C with gentle rotation. Cells were seeded at 5 × 10^4 ^per well on FN- or LN-coated slides (Lab Tek) and incubated for 2 h at 37°C. Immunofluorescence staining was preformed as described previously [42]. Briefly, cells were fixed in 3.7% paraformaldehyde for 30 min and then, permeablized with 0.3% Triton X-100 for 15 min. The slides were blocked with 1% BSA for 30 min, incubated with primary antibodies against integrin β_1 _(clone JB1B at 1:50; Santa Cruz), FAK-pY397 (MAB1144 at 1:50 dilution; Millipore), and paxillin-pY118 (at 1:200, Cell Signaling) overnight at 4°C, and then with FITC- or Cy3-conjugated secondary antibodies (at 1:200; Jackson ImmunoResearch Laboratories, West Grove, PA) for 1 h at room temperature. In some cases, the slides were further stained with Oregon Green 488-phalloidin (at 1:40; Invitrogen) for 30 min. After optimization of the immunofluoresence staining, each test was performed in triplicates and repeated three times independently.

## Mass-spectrophotometric analysis of sphingolipids

Subconfluent culture plates were washed twice with PBS, and incubated in their basal medium for 24 h. After washing the plates twice with ice-cold PBS, cells were scraped, centrifuged, and cellular Cer levels was measured by matrix-assisted laser desorption mass spectrometry (MALDI-MS) which included a panel of C14 to C26 Cer species: sphingomyeline, sphingosine, sphingosine-1-phosphate (S-1-P), and the dihydro analogues of sphingosine and S-1-P. The assay was performed in duplicate and repeated two times independently. Cer content was quantitated and calibrated to the intracellular phosphate (Pi) level and depicted as Cer (pM)/Pi (nM).

## Ceramide treatment

Cell permeable bioactive N-Hexanoyl-D-erythro-sphingosine (C6-*D-e*-Cer), inactive N-Hexanoly-*L-erythro*-sphingosine (C6-*L-e*-Cer), and N-Hexanoly-*D-threo*-sphingosine (C6-*D-t*-Cer) were purchased from Matreya, LLC (Pleasant Gap, PA). To determine the effect of Cer on β_1A_-integrin expression, cells were treated with active or inactive Cer analog at 8 to 32 μM for 36 h in complete medium and then, for 24 h in basal medium before immunoblotting. The effect of Cer on cell adhesion, migration, and invasion was determined by treating cells with 1 or 2 μM of active or inactive Cer for 5 days followed by 24 h incubation in basal medium before the functional assays. The effect of Cer on cell growth was measured by MTS assay as described in cell proliferation assay. Cytotoxicity of Cer was determined in parallel experiments using trypan blue exclusion assay.

### Statistical analyses

Data were analyzed using SAS v9.1 (SAS Institute, Cary, NC). Various ANOVA models were used. Nesting of assayed biological specimens (clones) in treatments were accounted for, and included as random effects. Post-hoc comparisons were performed with the Dunnett or Tukey procedures. Two-sample *t*-tests with Satterthwaite correction, when needed, were used to compare experimental settings with two groups. Cell counts were logarithm transformed as necessary before ANOVA was completed. An experiment-wise significance level of 0.05 was used; similarly, simultaneous confidence intervals of means were obtained.

## Competing interests

The authors declare that they have no competing interests.

## Authors' contributions

SK as principle investigator conceived the ideas, trained his research team for various techniques including cell adhesion, migration, and invasion assays, performed the initial studies described in the manuscript, coordinated the experiments, and supervised the entire project in his laboratory where all experiments were carried out. SK and SH were involved in the design and execution of the experiments, analyzing data and interpretation, and writing the manuscript. SH performed most of the experiments described in various sections of the manuscript including establishment and characterization of the stable PSAP-KD cells, transient transfection studies, protein and gene expression assays, cell adhesion, migration, and invasion assays, experiments on PSAP and Cer, and determining cathepsin D expression and proteolytic activity. ND performed the pilot studies to differentiate PSAP-KD cells from control transfectants. ZL was responsible for routine cell culture and assisted SH on some of the experiment on cell adhesion and immunoblotting. TL was partially involved in protein expression analysis for integrins. JG was involved in cell culture maintenance and performed part of the experiments on immunoprecipitation. AP provided training and expertise on immunofluorescence staining, microscopic data collection, and analysis. CV-G was responsible for all statistic analysis. All authors read and approved the manuscript.

## Supplementary Material

Additional file 1 **PSAP expression in three different prostate cancer progression models**. Cells were cultured in their complete media up to 75% confluency, washed with PBS, and incubated in their respective basal media for 24 h. Cell extracts and concentrated culture supernatants were prepared as described in the Materials and methods section. Equal amount of protein samples (15 μg cell extracts or concentrated culture supernatants) were resolved by SDS-PAGE under reducing conditions and subjected to Western analysis using a mouse monoclonal antibody against human saposin C. The GAPDH antibody was used for protein loading. Pr. Ep, normal human prostate epithelial cells; C4-2B, a bone metastatic AI-subline of androgen-sensitive LNCaP; p69, a human normal prostate epithelial cells immortalized with SV40 T-antigen; M12, a metastatic subline of p69 cell line; M2182, a highly invasive and metastatic subline of M12 cell line; PC-3M, a highly metastatic subline of PC-3 cells. Click here for file

Additional file 2 **Expression and purification of rhPSAP in CHO-K1 cells**. The cDNA of the human PSAP gene (NM_002778) was tagged with c-terminal 6×histidine by PCR amplification with the following primers 5'-AAA GCG GCC CAG CCG GCC GGC CCG GTC CTT GGA CTG-3' (forward) and 5'- CCG CTC GAG CTA GTG ATG GTG ATG GTG ATG GTT CCA CAC ATG GCG TTT GC-3' (backward). The PCR product was digested with the restriction enzymes *SfiI *and *XhoI *(New England Biolabs) and sub-cloned into a mammalian expression vector pSectag2A (Invitrogen). The positive clones were selected and confirmed by DNA sequencing. CHO-K1 (ATCC) cells were cultured in F12-K medium supplemented with 10% FBS and 1% penicillin-streptomycin. Cells (3 × 10^5^) were seeded in 60 mm dishes and cultured overnight to 20-30% confluency. Cells were transfected with 4 μg DNA of the pSectag2A/rhPSAP-His_6 _vector and 20 μl Lipofectin (Invitrogen) for 16 h. Several stable clones were isolated after selection with 500 μg/ml of Zeocin for two weeks. One stable clone with the highest rhPSAP expression was used for large-scale, rhPSAP purification. The stable cells were cultured in several T500 Triple layer flasks (Nunc) up to 90% confluency and replaced with 60 ml of OptiMEM medium (Invitrogen). After 48 h, the culture medium was pooled for centrifugation at 500 × g for 10 min at 4°C and the cleared supernatant was filtered with a 0.22 μm membrane. One liter of the supernatant was mixed with 5 ml Ni-NTA Superflow Resins (Qiagen) and incubated for 4 h at room temperature or overnight at 4°C by slow gyroscopic spin at 100 rpm. The resins were washed with the gradient imidazole at 10, 20, 50, and 100 mM in a binding buffer (50 mM Na_2_HPO_4_, pH 8.0, 300 mM NaCl). The appropriate fractions which contained the purified rhPSAP protein were pooled, and the buffer was exchanged to PBS by ultrafiltration using Vivaspin concentrators with a 10 KDa cut-off membrane. The purified rhPSAP were quantified by measuring OD280/260 nm; then they were filter-sterilized, and stored at -80°C for future use. Click here for file

Additional file 3 **Coomassie blue staining of the purified rhPSAP protein**. Recombinant proteins expressed in 1 liter of culture supernatants were purified by using Ni-NTA Superflow resin and polypropylene purification column. Forty μl of culture supernatant, 20 μl of imidazole washing, or elution fraction of CHO-K1 stable transfectants (clone # 2-2) were mixed with a non-reducing sample-loading buffer and separated in 4-20% Tris-Glycine gel. Solid arrows indicate imidazole-eluted rhPSAP (~68-72 kDa). Click here for file

Additional file 4 **Silver staining and western analysis of purified rhPSAP**. Recombinant PSAP proteins were subjected to SDS-PAGE using a 10% Tris-Glycine gel. The gels were subjected to silver staining and western blotting with an anti-PSAP antibody as indicated in the Materials and Methods section. Both silver staining and immunoblotting showed the presence of the same molecule with the expected molecular weight for PSAP. Click here for file

Additional file 5 **Biological activity of the purified rhPSAP**. The effect of the rhPSAP protein on prostate stromal cells was determined in an in vitro migration assay. Briefly, cells were seeded in the upper compartment of transwell filters in a basal medium supplemented with 0.1% BSA. The lower compartment was filled with 400 μl of either 5% FBS (as a positive control) or basal media supplemented with 0.1% BSA with or without rhPSAP at the indicated concentrations. After 24 h of incubation, the cells were fixed and stained with Diff-Quick. Non-migrated cells were removed by a cotton swap and the total cell number per filter was counted. Each sample was assayed in quadruplicates. Data represented the average of three independent experiments ± SEM. Statistical significance (*p *< 0.05) between the control and treatment groups was evaluated by one-way ANOVA test with Bonferroni adjustment. Click here for file

Additional file 6 **PSAP down modulation decreased FAK binding to Src and Paxillin phosphorylation in metastatic PCa cells**. PSAP-KD cells were incubated in their basal medium for 24 h and whole cell lysates were extracted and subjected to immunoprecipitation for Src and immunoblotting with anti-FAK or-paxillin antibody as described in the Materials and Methods section. Similar data were obtained from three independent experiments. Click here for file

## Achnowledgements

We gratefully acknowledge Dr. Lucia R. Languino from University of Massachusetts for providing antibodies against β_1B _and β_1C _integrin isoforms. We thank Dr. J. L. ware from the Department of Pathology, Medical College of Virginia for providing the p69, M12, and M2182 cell lines. We thank Ms. Jonna Ellis and Ms. Heidi L. Davis at Stanley S. Scott Cancer Center, LSUHSC for editing the manuscript. S.K. was funded by Stanley S. Scott Cancer Center (S.K), Louisiana Cancer Research Consortium (S.K), Louisiana State University Health Sciences Center and Tulane University Clinical and Translational Research Education and Commercialization Project (CTRECP-070; to SK), NIH-NCRR; 1P20 RR021970 (A. Ochoa/S.K), and NCI 1R21 CA120625 (to S.K.).
