# Supplementary figures and images for: Prosaposin down-modulation decreases metastatic prostate cancer cell adhesion, migration, and invasion
Source: Mol Cancer. 2010 Feb 4;9:30. doi: 10.1186/1476-4598-9-30 (PMC2825248; doi:10.1186/1476-4598-9-30)

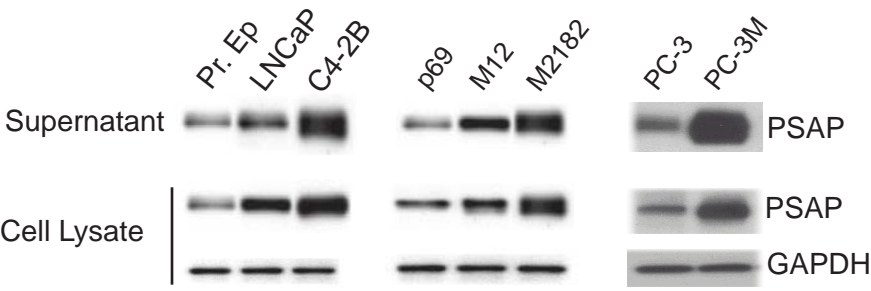

Supplement: Additional file 1 — PSAP expression in three different prostate cancer progression models. Cells were cultured in their complete media up to 75% confluency, washed with PBS, and incubated in their respective basal media for 24 h. Cell extracts and concentrated culture supernatants were prepared as described in the Materials and methods section. Equal amount of protein samples (15 μg cell extracts or concentrated culture supernatants) were resolved by SDS-PAGE under reducing conditions and subjected to Western analysis using a mouse monoclonal antibody against human saposin C. The GAPDH antibody was used for protein loading. Pr. Ep, normal human prostate epithelial cells; C4-2B, a bone metastatic AI-subline of androgen-sensitive LNCaP; p69, a human normal prostate epithelial cells immortalized with SV40 T-antigen; M12, a metastatic subline of p69 cell line; M2182, a highly invasive and metastatic subline of M12 cell line; PC-3M, a highly metastatic subline of PC-3 cells. [file 1476-4598-9-30-S1.PDF]

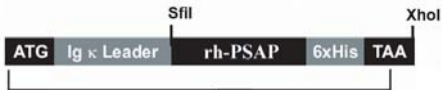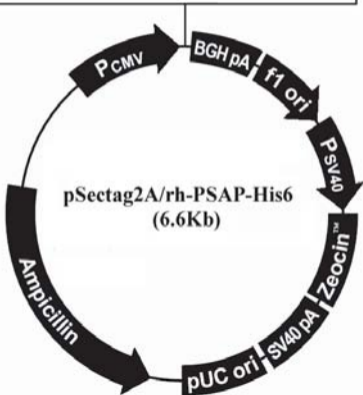

Supplement: Additional file 2 — Expression and purification of rhPSAP in CHO-K1 cells. The cDNA of the human PSAP gene (NM_002778) was tagged with c-terminal 6×histidine by PCR amplification with the following primers 5'-AAA GCG GCC CAG CCG GCC GGC CCG GTC CTT GGA CTG-3' (forward) and 5'- CCG CTC GAG CTA GTG ATG GTG ATG GTG ATG GTT CCA CAC ATG GCG TTT GC-3' (backward). The PCR product was digested with the restriction enzymes SfiI and XhoI (New England Biolabs) and sub-cloned into a mammalian expression vector pSectag2A (Invitrogen). The positive clones were selected and confirmed by DNA sequencing. CHO-K1 (ATCC) cells were cultured in F12-K medium supplemented with 10% FBS and 1% penicillin-streptomycin. Cells (3 × 10^5^) were seeded in 60 mm dishes and cultured overnight to 20-30% confluency. Cells were transfected with 4 μg DNA of the pSectag2A/rhPSAP-His6 vector and 20 μl Lipofectin (Invitrogen) for 16 h. Several stable clones were isolated after selection with 500 μg/ml of Zeocin for two weeks. One stable clone with the highest rhPSAP expression was used for large-scale, rhPSAP purification. The stable cells were cultured in several T500 Triple layer flasks (Nunc) up to 90% confluency and replaced with 60 ml of OptiMEM medium (Invitrogen). After 48 h, the culture medium was pooled for centrifugation at 500 × g for 10 min at 4°C and the cleared supernatant was filtered with a 0.22 μm membrane. One liter of the supernatant was mixed with 5 ml Ni-NTA Superflow Resins (Qiagen) and incubated for 4 h at room temperature or overnight at 4°C by slow gyroscopic spin at 100 rpm. The resins were washed with the gradient imidazole at 10, 20, 50, and 100 mM in a binding buffer (50 mM Na2HPO4, pH 8.0, 300 mM NaCl). The appropriate fractions which contained the purified rhPSAP protein were pooled, and the buffer was exchanged to PBS by ultrafiltration using Vivaspin concentrators with a 10 KDa cut-off membrane. The purified rhPSAP were quantified by measuring OD280/260 nm; then they were filter-sterilized, an [file 1476-4598-9-30-S2.PDF]

rh-PSAP  
(pSectag2A-His)

KDa

160 —  
110 —  
80 —  
60 —  
50 —  
40 —  
30 —  
20 —  
15 —  
10 —

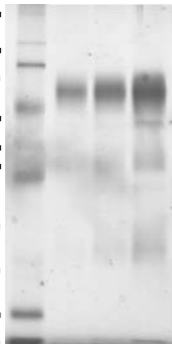

Silver staining

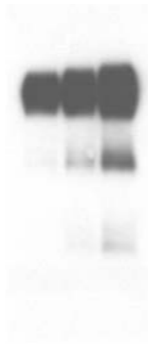

← rh-PSAP

Western Blot

Supplement: Additional file 4 — Silver staining and western analysis of purified rhPSAP. Recombinant PSAP proteins were subjected to SDS-PAGE using a 10% Tris-Glycine gel. The gels were subjected to silver staining and western blotting with an anti-PSAP antibody as indicated in the Materials and Methods section. Both silver staining and immunoblotting showed the presence of the same molecule with the expected molecular weight for PSAP. [file 1476-4598-9-30-S4.PDF]

# Prostate Stromal Cells

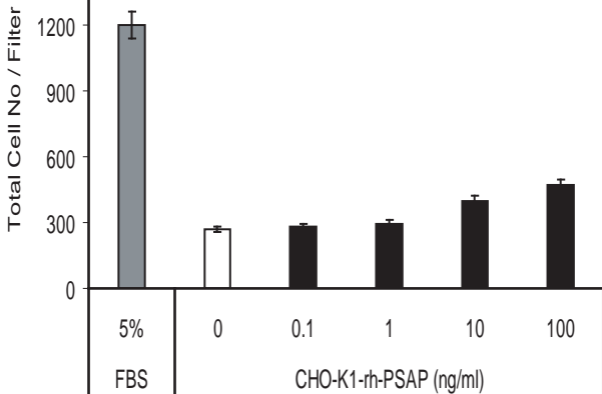

Supplement: Additional file 5 — Biological activity of the purified rhPSAP. The effect of the rhPSAP protein on prostate stromal cells was determined in an in vitro migration assay. Briefly, cells were seeded in the upper compartment of transwell filters in a basal medium supplemented with 0.1% BSA. The lower compartment was filled with 400 μl of either 5% FBS (as a positive control) or basal media supplemented with 0.1% BSA with or without rhPSAP at the indicated concentrations. After 24 h of incubation, the cells were fixed and stained with Diff-Quick. Non-migrated cells were removed by a cotton swap and the total cell number per filter was counted. Each sample was assayed in quadruplicates. Data represented the average of three independent experiments ± SEM. Statistical significance (p < 0.05) between the control and treatment groups was evaluated by one-way ANOVA test with Bonferroni adjustment. [file 1476-4598-9-30-S5.PDF]

PC-3

DU-145

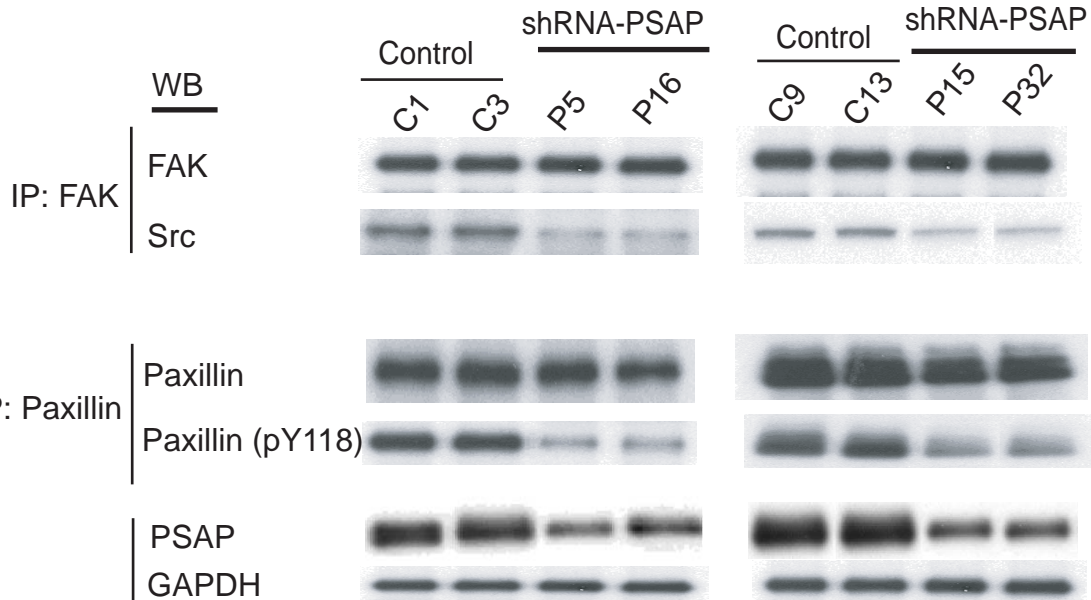

Supplement: Additional file 6 — PSAP down modulation decreased FAK binding to Src and Paxillin phosphorylation in metastatic PCa cells. PSAP-KD cells were incubated in their basal medium for 24 h and whole cell lysates were extracted and subjected to immunoprecipitation for Src and immunoblotting with anti-FAK or-paxillin antibody as described in the Materials and Methods section. Similar data were obtained from three independent experiments. [file 1476-4598-9-30-S6.PDF]
