# Supplementary material for: Prosaposin down-modulation decreases metastatic prostate cancer cell adhesion, migration, and invasion
Source: Mol Cancer. 2010 Feb 4;9:30. doi: 10.1186/1476-4598-9-30 (PMC2825248; doi:10.1186/1476-4598-9-30)
Supplement: Additional file 3 — Coomassie blue staining of the purified rhPSAP protein. Recombinant proteins expressed in 1 liter of culture supernatants were purified by using Ni-NTA Superflow resin and polypropylene purification column. Forty μl of culture supernatant, 20 μl of imidazole washing, or elution fraction of CHO-K1 stable transfectants (clone # 2-2) were mixed with a non-reducing sample-loading buffer and separated in 4-20% Tris-Glycine gel. Solid arrows indicate imidazole-eluted rhPSAP (~68-72 kDa). [file 1476-4598-9-30-S3.PDF]

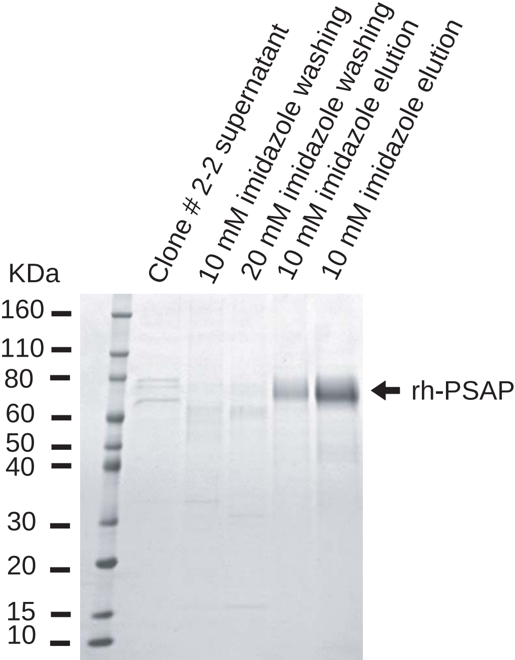

Clone # 2-2 supernatant
10 mM imidazole washing
20 mM imidazole washing
10 mM imidazole elution
10 mM imidazole elution
KDa
160
110
80
60
50
40
30
20
15
10
rh-PSAP
